# Serial image interpretation tasks improve accuracy and increase confidence in Level 1 echocardiography reporting: a pilot study

**DOI:** 10.1186/s44156-023-00018-9

**Published:** 2023-04-06

**Authors:** Richard Fisher, Amal Zayan, Jennifer Gosling, Joao Ramos, Mahmoud Nasr, David Garry, Alexandros Papachristidis, Francisca Caetano, Philip Hopkins

**Affiliations:** 1 Department of Critical Care, King’s College Hospital NHS Foundation Trust, London, UK; 2 North-West London School of Anaesthesia / Pan-London School Intensive Care Medicine, London, UK; 3 Department of Critical Care, Oxford University Hospitals NHS Foundation Trust, Oxford, UK; 4 Department of Cardiology, King’s College Hospital NHS Foundation Trust, London, UK; 5 Department of Critical Care, Royal Papworth Hospital NHS Foundation Trust, Cambridge, UK

**Keywords:** Echocardiography, Focused echocardiography, Point-of-care, Ultrasound, Pocus, Image interpretation, Skill acquisition, Medical education, Distance education

## Abstract

**Background:**

Focused echocardiography is increasingly used in acute and emergency care, with point-of-care ultrasound integrated into several specialist training curricula (e.g. Emergency Medicine, Cardiology, Critical Care). Multiple accreditation pathways support development of this skill but there is scant empirical evidence to inform selection of teaching methods, accreditation requirements or quality assurance of education in focussed echocardiography. It has also been noted that access to in-person teaching can be a barrier to completing accreditation programmes, and that this may affect learners disproportionately depending on the location or nature of their institution. The purpose of the study was to determine whether serial image interpretation tasks as a distinct learning tool improved novice echocardiographers’ ability to accurately identify potentially life-threatening pathology from focused scans. We also aimed to describe the relationship between accuracy of reporting and participants’ confidence in those reports, and to assess users’ satisfaction with a learning pathway that could potentially be delivered remotely.

**Methods:**

27 participants from a variety of healthcare roles completed a program of remote lectures and 2 in-person study days. During the program they undertook 4 ‘packets’ of 10 focused echocardiography reporting tasks (total = 40) based on images from a standardised dataset. Participants were randomized to view the scans in varying orders. Reporting accuracy was compared with consensus reports from a panel of expert echocardiographers, and participants self-reported confidence in their image interpretation and their satisfaction with the learning experience.

**Results:**

There was a stepwise improvement in reporting accuracy with each set of images reported, from an average reporting score of 66% for the 1st packet to 78% for the 4th packet. Participants felt more confident in identifying common life-threatening pathologies as they reported more echocardiograms. The correlation between report accuracy and confidence in the report was weak and did not increase during the study (r_s_ = 0.394 for the 1st packet, r_s_ = 0.321 for the 4th packet). Attrition during the study related primarily to logistical issues. There were high levels of satisfaction amongst participants, with most reporting that they would use and / or recommend a similar teaching package to colleagues.

**Conclusions:**

Healthcare professionals undertaking remote training with recorded lectures, followed by multiple reporting tasks were capable of interpreting focused echocardiograms. Reporting accuracy and confidence in identifying life-threatening pathology increased with the number of scans interpreted. The correlation between accuracy and confidence for any given report was weak (and this relationship should be explored further given the potential safety considerations). All components of this package could be delivered via distance learning to enhance the flexibility of echocardiography education.

**Supplementary Information:**

The online version contains supplementary material available at 10.1186/s44156-023-00018-9.

## Background

In the United Kingdom (UK), Point-Of-Care UltraSound (POCUS) including focused (Level 1) echocardiography is now a component of the training curricula for doctors training in cardiology, emergency medicine and intensive care medicine [1–3]. Competence can be demonstrated by completion of one of a number of accreditation processes [4]. As an example, the British Society of Echocardiography (BSE) Level 1 accreditation requires healthcare professionals to demonstrate their ability to generate and interpret echocardiography images through the completion of a logbook (≥ 75 cases) and an examination (held nationally, twice a year). To pass the examination individuals need to be able to reliably identify common life-threatening pathologies [5].

The skills required to generate echocardiographic images, and to interpret these images (identifying significant pathology) are discrete. Evidence suggests that these skills are acquired at different rates, with interpretation taking longer [6].

This is exemplified by the pass rates of candidates undertaking the BSE Level 1 practical exam. In the first 9 sittings of the practical exam, 69 individual candidates attempted the exam, with 90% passing the image acquisition station at the first attempt, and 84% passing the video cases station at the first attempt (unpublished data).

At our institution image interpretation tasks are one component of an educational package used to train healthcare professionals in Level 1 echocardiography. The purpose of this study was to evaluate the impact of performing multiple image interpretation tasks (in isolation from other components of teaching) on objective reporting accuracy and subjective confidence amongst novice echocardiographers. In addition, we aimed to assess learners’ satisfaction with this program as an educational tool.

## Methods

Four members of the critical care team (AZ, JG, JR, MN) produced a library of 40 focused echocardiograms covering the BSE Level 1 syllabus (including ‘normal’ studies, which do not demonstrate any pathology). All studies were recorded during routine patient management and anonymized as per the Royal College of Radiologist’s guidance [7]. Video clips and still images for each echocardiogram were edited into a single video file with each view being looped for around 10s. Only views and measurements that feature in the BSE Level 1 minimum dataset were included [8]. The average video length was 182s (range 85–240s).

Studies were then independently reviewed by two experts in critical care echocardiography (RF, DG), and a standardised reporting template used to describe 15 components of the echocardiogram using ‘best fit’ multiple-choice answers (Additional file 1: Figure S1). When the answers selected by the two reviewers were not identical (for example one reviewer may have felt mitral regurgitation was mild and hence ‘not significant’, whilst the other felt it was moderate and therefore described it as ‘significant’) two further expert reviewers (AP, FC) adjudicated. The additional reviewers could decide that one of the suggested answers should be considered correct, or that there was enough uncertainty that either would be accepted. In addition, the initial two reviewers (RF, DG) scored each of the 15 components between 1 and 3, with a score of 3 being awarded for a critical finding that could have a serious negative impact on patient outcome if missed, 2 for major findings that were not felt to be immediately life-threatening, and 1 for minor findings. This scoring system meant that each echocardiogram had a different number of maximum available marks (average available marks 44, range 30–58).

All authors producing or reviewing the library hold relevant echocardiography accreditations: RF, AP and FC hold the European Association of CardioVascular Imaging (EACVI) Level 2 TTE accreditation; DG holds the BSE Level 2 TTE accreditation; AZ, JG, JR and MN hold the BSE Level 1 TTE accreditation. RF is the current BSE lead for Level 1 echocardiography. DG is the immediate past BSE lead for Level 1 echocardiography. All authors are actively involved in the provision of training in focused echocardiography.

The 40 echocardiograms were randomly assigned to one of four packets (A1, A2, B1, B2) using an online random number generator, such that each packet contained 10 echocardiograms.

Participants were invited to enroll in the study. The study was open to medical students, doctors of all grades, nurses and advanced critical care practitioners at our institution. As we wished to assess the impact on novice echocardiographers, we excluded individuals who had personally performed > 20 echocardiograms.

When participants enrolled, they were assigned a unique participant number used on all study paperwork. Participants were asked to watch a series of 7 online video lectures (total duration ~ 5 h), which provided instruction on how to approach interpreting focused echocardiograms [9].

A total of 8 study days were held, and participants each attended two study days (one during week 1 of the study, and one during week 2). Each study day included a morning and afternoon session. During each session participants would report the 10 echocardiograms within a single packet, presented in a random order (created using an online random number generator). Additional file 2: Figure S2 shows the different potential sequences in which participants could view the echocardiograms.

During sessions, participants viewed a looped video of the echocardiogram 3 times, while completing a standardised reporting template (Additional file 1: Figure S1). They were also asked to rate their subjective confidence in the accuracy of their report, by placing a vertical line through visual analogue scale 100 mm in length (where a mark of 0 mm reflected no confidence in the report, and a mark of 100 mm reflected complete confidence). Immediately following submission of their report, participants would be shown the echocardiogram again, alongside details of critical and major findings as agreed by the expert reviewers, and an audio description of the echocardiogram highlighting key pathologies (narrated by RF). After every packet of 10 echocardiograms had been reviewed, participants had the opportunity to discuss the images with the study team and ask for points of clarification. Over the course of the study days all participants reported each of the 40 echocardiograms once.

The total marks achieved for each report were summed and converted to a percentage of the total available marks for that echocardiogram. For each of the 40 echocardiograms the mean percentage score obtained across all participants was calculated. Each individual participant’s score was then expressed as a proportion of the average score (with a score < 1 representing the participant had performed less well than average when reporting this specific echocardiogram, and a score > 1 representing the participant had performed better than average). Following this the participants average score for all the echocardiograms within a single packet was averaged (we termed this Participant Packet Difference—PPD—with a score < 1 representing the participant had performed less well than average when considering the 10 echocardiograms within this specific packet, and a score > 1 representing the participant had performed better than average).

The primary outcome was the change in PPD from the first packet attempted (timepoint 1), to each of the three subsequently attempted packets (timepoints 2, 5 and 6).

We asked participants to rate their subjective confidence in reporting focused echocardiograms at the start and end of each of their two study days. Participants rated their confidence using a visual analogue scale 100 mm in length (where a mark of 0 mm reflected no confidence, and a mark of 100 mm reflected maximal confidence). Participants rated their confidence with respect to their ability to identify:

- immediately life-threatening pathology
- significant left ventricular impairment
- significant right ventricular impairment
- significant valvular incompetence
- significant pericardial effusions

Secondary outcomes included: change in subjective confidence from the start of the first study day (timepoint 0), to the end of the first and second study days (timepoints 3 and 7 respectively), the correlation between report accuracy (expressed as a percentage of available marks) and subjective confidence in that report (stratified by packet, timepoints 1, 2, 5 and 6).

At the end of the second study day (timepoint 7) participants used a visual analogue scale 100 mm in length to rate their satisfaction with the process of completing serial image interpretation tasks, their likelihood of recommending this process to other novice echocardiographers and their own likelihood to access an online library of similar studies and reporting tasks, if one were to be developed.

### Statistical analysis

Statistical analysis was performed by RF. For the primary outcome a two-tailed paired t-test was used to compare the PPD obtained at timepoints 2, 5 and 6 (the second, third and fourth packets attempted respectively) against timepoint 1 (the first packet attempted). For the secondary outcome of change in subjective confidence, a two-tailed paired t-test was used to compare timepoints 3 and 7 (end of day 1 and day 2 respectively) against timepoint 0 (start of day 1). For the secondary outcome of correlation between report accuracy and subjective confidence, results were stratified by packet attempted (timepoints 1, 2, 5 and 6) and a Spearman rank correlation test was used to assess correlation.

## Results

48 participants registered to take part in the study (numbers capped due to classroom size). Of these 12 did not attend the first study day (5 could not secure study leave, 2 cited acute illness, 1 required carer’s leave, 4 did not cite a reason), and a further 9 participants attended their first study day, but not their second (2 cited acute illness, 1 required carer’s leave, 6 did not cite a reason). Final analysis was conducted based on the results from the 27 participants who attended two study days, and each attempted to report all 40 prepared echocardiograms.

Of the 27 participants 18 (66.7%) were doctors (1 in the Foundation program, 12 core trainees, 4 specialty trainees, 1 consultant), 5 (18.5%) were nurses, 3 (11.1%) were qualified or trainee Advanced Critical Care Practitioners (ACCP) and 1 (3.7%) an under-graduate medical student. 15 participants (55.6%) were female. 9 participants (33.3%) had previously attended a hands-on focused echocardiography course. Most (15, 55.6%) had never performed an echocardiogram previously, whilst 9 (33.3%) had previously performed between 1 and 10 scans, and 3 (11.1%) had performed between 11 and 20 scans.

All 27 participants attempted to report all 40 echocardiograms. Details of the echocardiograms can be found in Additional file 4: Table S1.

**Fig. 1:**
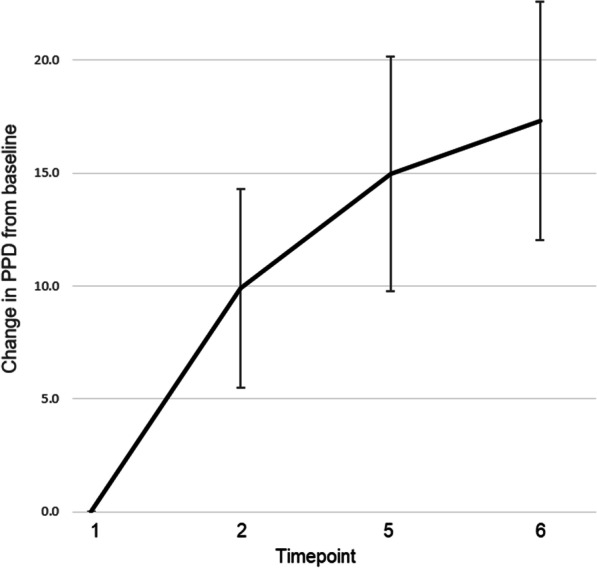
Primary outcome: Change in Participant Packet Difference (PPD) from baseline

The mean PPD for the first packet attempted was − 10.5 (i.e. on average participants attained 89.5% of the average mark, when attempting their first through tenth image interpretation tasks). This rose to -0.7 for the second packet attempted, + 4.4 for the third packet attempted and + 6.8 for the fourth packet attempted. The absolute change in PPD from baseline (timepoint 1) at the three subsequent timepoints (2, 5 and 6) was + 9.9 (95%CI 5.5–14.3, p = 0.0001), + 15.0 (95%CI 9.8–20.2, p < 0.00001) and + 17.3 (95%CI 12.0–22.6, p < 0.00001) respectively (Fig. 1) (individual participant change in PPD from baseline can be seen in Additional file 3: Figure S3).

**Fig. 2:**
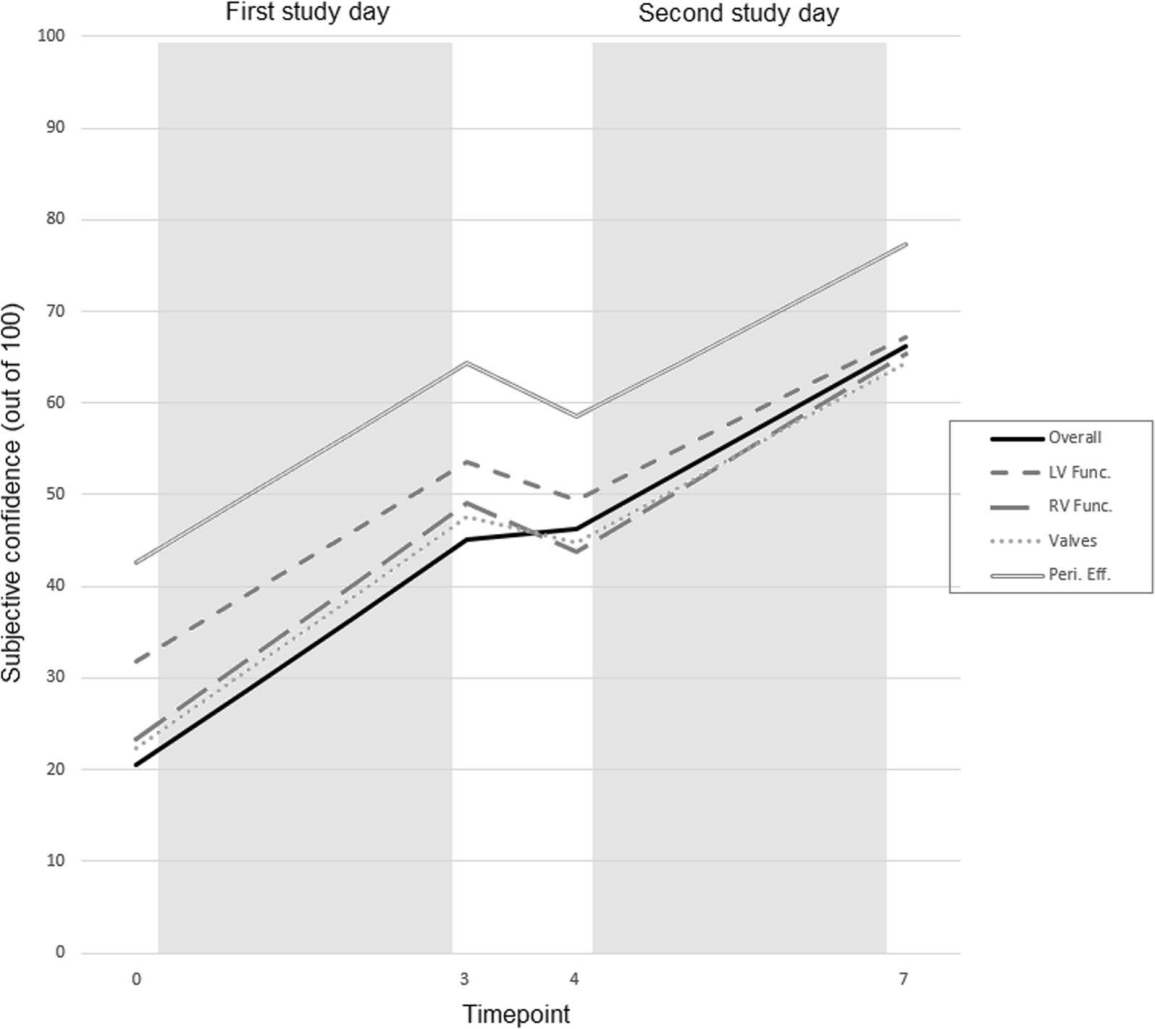
Subjective confidence (on a scale of 0–100)

For all measures of subjective confidence there was a significant rise from baseline (timepoint 0) to the end of both study days (timepoints 3 and 7) (Table 1 and Fig. 2).

**Table 1:** Subjective confidence in ability to recognise significant pathologies

	Baseline, %	End of Day 1, %	Change, % (± SD)	End of Day 2, %	Change, % (± SD)
Overall	20	45	25 (16)	66	46 (22)
LV impairment	32	54	22 (18)	67	35 (22)
RV impairment	23	49	26 (20)	65	42 (20)
Valvulopathies	22	48	26 (19)	64	42 (19)
Pericardial effusions	43	64	21 (24)	77	34 (26)

p-value < 0.0001 for all

**Fig. 3:**
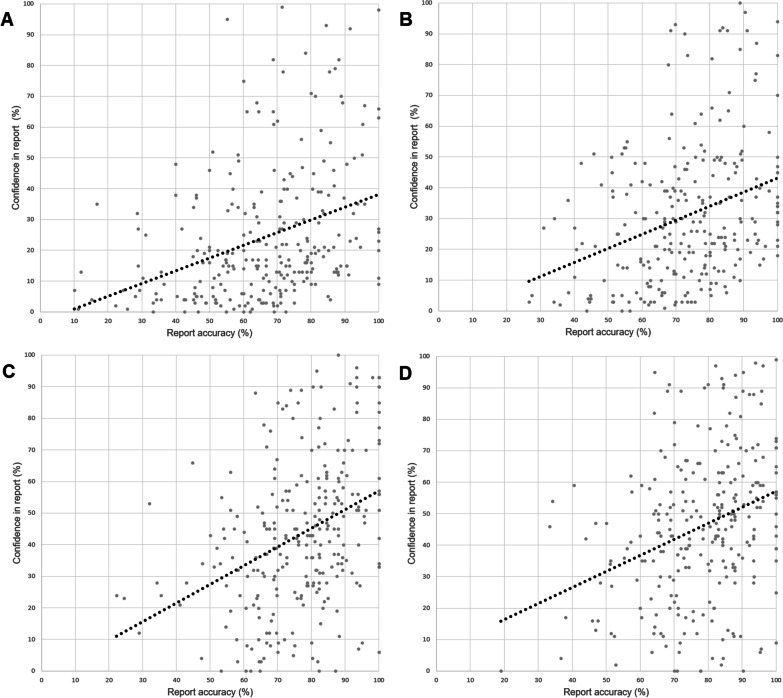
Correlation between report accuracy and subjective confidence in report. **A** first packet attempted (timepoint 1); n = 266*; mean reporting accuracy 66%; mean confidence in report 24%; line of best fit y = 0.4117x-3.0899; r_s_ = 0.394; p-value < 0.00001. **B** second packet attempted (timepoint 2); n = 259*; mean reporting accuracy 73%; mean confidence in report 31%; line of best fit y = 0.4558x-2.4703; r_s_ = 0.339; p-value < 0.00001. **C** third packet attempted (timepoint 5); n = 268*; mean reporting accuracy 77%; mean confidence in report 43%; line of best fit y = 0.5918x-2.1069; r_s_ = 0.392; p-value < 0.00001. **D** fourth packet attempted (timepoint 6); n = 266*; mean reporting accuracy 78%; mean confidence in report 46%; line of best fit y = 0.5105x + 6.0985; r_s_ = 0.321; p-value < 0.00001. *1080 echocardiograms reported, but subjective confidence only rated for 1059 echocardiograms (98.1%)

There was a weak correlation between report accuracy and subjective confidence in the report at all timepoints throughout the study (Fig. 3).

Twenty-three participants completed an end of study questionnaire. Mean satisfaction with the process of using image interpretation tasks to learn echocardiography was rated at 80% (SD ± 16%). Mean likelihood of recommending image interpretation tasks to others was rated at 84% (SD ± 14%). Mean likelihood to engage with an online library of similar image interpretation tasks was rated at 84% (SD ± 17%).

## Discussion

The main findings of this study are:

- Healthcare professionals undertaking remote training with recorded lectures were capable of interpreting Level 1 echocardiograms with an initial average report score of 66%.
- There was sequential improvement in report accuracy with an increasing number of completed reports.

Participants felt more confident in identifying common life-threatening pathologies as they reported more echocardiograms, but this did not correlate with more accurate reporting of any given scan.

Our data showed that healthcare professionals undertaking 40 Level 1 echocardiography image interpretation tasks had a sequential improvement in report accuracy as they completed a greater number of reports. The mean report score for all participants, for the first packet of 10 interpretation tasks, was 66%. This rose to 78% by the time participants were undertaking their fourth packet. We designed in compensation for the fact that some tasks may have been inherently ‘easier’ than others (the mean score achieved for different tasks ranged from 61.7% up to 88.9%), by making our primary outcome measure change in PPD from baseline based on the score achieved during the first attempted packet. This demonstrated a sequential increase reflecting improving accuracy as participants completed a greater number of tasks. The magnitude of this rise decreased during each step of the study. Whilst there was a significant increase in PPD between the first and second, and second and third packets, the rise between the third and fourth packets did not reach statistical significance (+ 2.3, p = 0.06). This suggests that by this stage we were already experiencing a ‘levelling off’ of benefit.

In addition, we found that participants felt more confident in identifying key pathologies as they performed more tasks, with mean initial confidence rated at 20% at the start of the first study day, and rising to 66% by the end of the second study day.

Interestingly, we also found that whilst reporting accuracy and overall confidence both increased during the study, the participants ability to predict the accuracy of any given report did not improve. We correlated the report accuracy and confidence in the report for each task (Fig. 3). Whilst at each timepoint there was a correlation between accuracy and confidence, the correlation was weak, and did not improve as participants completed more tasks. A similar finding was made by Zawadka et al. who carried out pre and post course knowledge tests around a one-day point-of-care ultrasound course for medical students. They found that post course students were more confident in their test answers, for those questions where marks were higher, but also for those questions where marks did not increase [10].

Development of any POCUS educational resource requires consideration of how it may affect users’ confidence in a way that could cause harm in clinical practice (e.g. if users become inappropriately confident, and this influences decision making by them or others), and our study is not exempt from this. Nevertheless, many professionals use POCUS in their practice without any specific credentialling [11, 12], and this is likely to increase as handheld US probes become less costly and there is a proliferation of free open access resources. While we believe that there are patient safety considerations attached to the ethical development of any educational resource, we do not suggest that completion of image interpretation tasks in isolation would be de facto evidence of competence or any form of accreditation. Furthermore, strategies to provide widespread access to learning resources that could be delivered remotely and asynchronously may improve the quality of educational material relied on by users who are not engaged with an accreditation pathway.

Whilst individuals were provided with an ideal answer immediately following each task, we did not provide individual feedback about the accuracy of their reports to participants during the course of the study, and doing this may have improved the correlation between confidence and accuracy. The authors also speculate that the number of tasks undertaken in this pilot may have been insufficient for participants to improve their ability to benchmark their own reporting accuracy. Given the potential safety implications this relationship should be explored in greater detail in any subsequent study.

In terms of the methods used, of 23 participants completing a satisfaction questionnaire at the end of the second study day, self-reported satisfaction with the tasks as a tool for learning echocardiography was high. Participants reported they would be likely to access an online library of similar tasks if one were created, and would recommend these tasks to their colleagues.

Our study has several strengths. Image interpretation tasks were taken from real-world practice and are therefore inherently clinically relevant. Participants were asked to review echocardiograms and provide focused reports, which is identical to the task they would be required to perform in clinical practice.

There are potential limitations to consider. Firstly, the number of participants was lower than we intended, with participants withdrawing either before or during the study (25.0% and 18.8% respectively). Of participants who attended the first study day, 6 (16.7%) did not attend the second study day and did not provide a reason for doing so. It is conceivable that these participants did not return for the second study day as they did not feel the first met their educational needs. Due to the relatively low participant numbers we felt it was not appropriate to complete sub-group analyses as planned in the original protocol. The participants had a vast range in prior clinical experience (ranging from a fourth-year medical student to a consultant in acute medicine), and it is not possible, based on our results, to determine if the increases in accuracy and confidence seen in our study can be expected uniformly across all groups.

The participants in this study all had little or no prior experience of reporting focused echocardiograms. We saw a rapid initial improvement, followed by a plateau. We cannot know what improvements would be seen in healthcare professionals with a greater baseline experience. Given that our novices were already seeing a drop off in improvement, it is conceivable that more experienced echocardiographers would need to complete many more tasks to demonstrate significant improvements in accuracy. There may be a maximum level of accuracy that can be obtained. In addition, there is also no consensus on what constitutes a ‘clinically significant’ improvement in this context.

In this study we included 40 tasks. This was a pragmatic decision, as we estimated the maximum number of tasks participants could undertake per day was ~ 20, and we felt that asking participants to commit to > 2 study days would hamper enrolment. In addition a study by Bowcock et al. found that healthcare professionals undertaking training in focused echocardiography, who had performed ≥ 40 focused echocardiograms previously, were able to detect abnormalities using an ultrasound simulator with 100% accuracy, whereas this rate was lower in professionals who had performed < 40 studies [6].

In this pilot study we ran 8 in-person study days in order to provide exposure to image interpretation tasks to 27 participants. Online learning modules including image libraries have been used in training healthcare professionals imaging including echocardiography [13, 14]. Indeed, the Royal College of Emergency Medicine explicitly describes ‘completing e-modules and quiz, reviewing image/video library with normal and abnormal sono-anatomy, including artifacts’ as a component of their POCUS pathway [2]. The authors propose that lessons learnt from this pilot study could inform the design of a larger study using an online library of cases. This would have a number of benefits. It would allow participants to work through tasks at their own rate, at a time convenient to them. This reduces some of the logistical barriers to POCUS training, which have been identified to adversely affect engagement with accreditation programs [11, 12, 15]. Flexible learning, where possible, may be particularly important in widening access to people with complex work schedules and specific responsibilities outside work as well as those whose attendance at in person training is limited by geography, time zones, financial constraints or disability. This is particularly important for those in a rotational training programme, where trainees’ experience of access to supervision for development of POCUS skills is markedly influenced by location [16]. Exposure to these tasks could be spread over a greater period of time (candidates attempting the BSE Level 1 accreditation will typically have performed studies over a period of up to 12 months), and learners could attempt a greater number of tasks. There is a precedent for ‘online asynchronous learning’ to increase learners’ clinical use in POCUS as well as their engagement with quality assurance review of their scanning and work towards credentialing [17]. Similar learning options reduce the attrition noted specifically in relation to focused echocardiography accreditation in the UK and internationally [11, 12, 15, 18].

Higher numbers of participants and tasks attempted would provide insight into different patterns of learning by which sub-groups develop image interpretation skills, as well as whether different components of image interpretation are learnt at different rates (for example do learners become proficient at recognising ventricular impairment sooner than recognising valvular incompetence?). Different focused echocardiography accreditation programs are widely variable in the minimum number of scans required to demonstrate competence (ranging from 10 to 100) [19]. The number of scans learners must perform is an arbitrary number derived from expert consensus opinion and is not backed-up by published data [20]. A better understanding of how quickly individuals develop accurate image interpretation could be useful in informing the design of accreditation pathways.

Finally the authors wish to stress that they believe image libraries and image interpretation tasks form only one component of studying focused echocardiography. The currently available accreditation pathways typically involve attendance at an in-person event, with a combination of hands-on teaching and lectures, followed by performance and reporting of a number of scans. The method of final assessment may be informal (‘sign-off’ by a local mentor—e.g. FUSIC Heart) or formal (attendance at a nationally or internationally organized examination—e.g. BSE Level 1, European Diploma in Advanced Critical Care Echocardiography) (4). Since the Covid-19 pandemic, some pathways have offered the option to complete the initial information based teaching online (e.g. FUSIC).

We do not propose that image libraries or interpretation tasks should necessarily replace any pre-existing teaching methods, but that they might serve as an important addition; providing additional interpretation practice and offering feedback to inform individuals’ perception of their performance. This may off-set the potential harms from use of focused echocardiography without completing a formal accreditation. Whilst this study did not set out to evaluate any other teaching/learning techniques, it seems likely that would-be echocardiographers will need to engage with a variety of different learning methods during their training, including (but not necessarily limited to): dedicated practical courses; hands-on bedside teaching (with real-time feedback); departmental education sessions; private study (including textbooks, guidelines and recorded lectures). Further research is required to understand the ideal composition of an integrated training program, and the relative importance of the component parts for different learners.

## Conclusion

Performing Level 1 echocardiography image interpretation tasks, combined with immediate feedback resulted in improved reporting accuracy and increased subjective confidence amongst 27 healthcare professionals with little or no previous experience of performing echocardiograms. We believe that these findings support development of online image libraries and related resources which could be accessed by learners wishing to develop this skill.

## Supplementary Information

**Additional file 1: Figure S1.** Study reporting template.

**Additional file 2: Figure S2.** Participant timeline.

**Additional file 3: Figure S3**. Change in Participant Packet Difference (PPD) from baseline for individual participants.

**Additional file 4: Table S1.** Summary of echocardiograms included in the study. AoV, Aortic Valve; AR, Aortic Regurgitation; LV, Left Ventricle; MR, Mitral Regurgitation; RV, Right Ventricle; RWMA, Regional Wall Motion Abnormality; TR, Tricuspid Regurgitation.

## Data Availability

The data that support the findings of this study are available from the authors upon reasonable request and with permission from the Critical Care Department at King's College Hospital.
