# Supplementary figures and images for: Serial image interpretation tasks improve accuracy and increase confidence in Level 1 echocardiography reporting: a pilot study
Source: Echo Res Pract. 2023 Apr 6;10:6. doi: 10.1186/s44156-023-00018-9 (PMC10076813; doi:10.1186/s44156-023-00018-9)

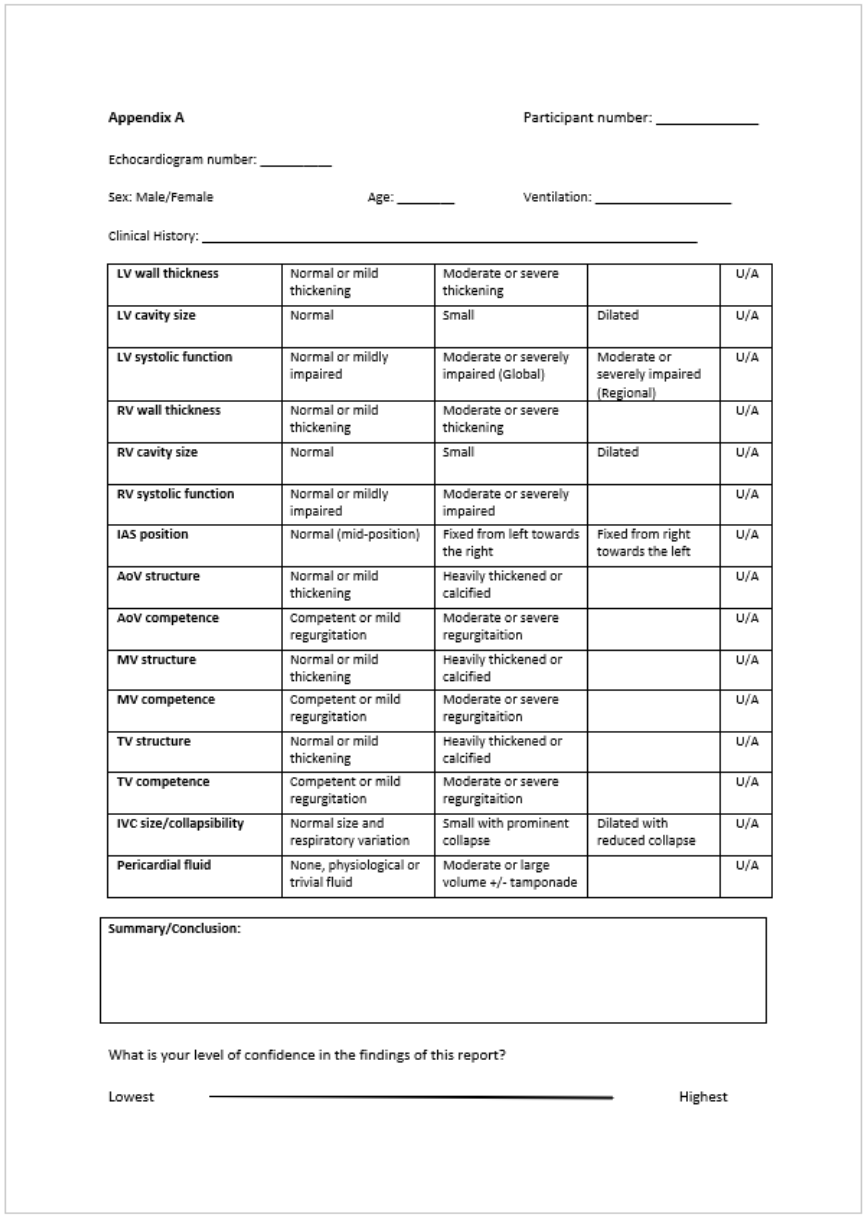

Supplement: Supplementary file 1 — Additional file 1: Figure S1. Study reporting template. [file 44156_2023_18_MOESM1_ESM.bmp]

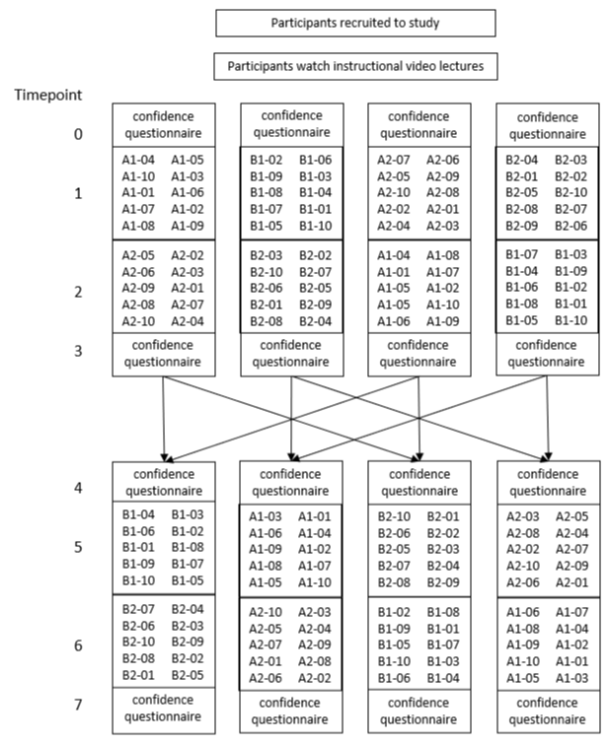

Supplement: Supplementary file 2 — Additional file 2: Figure S2. Participant timeline. [file 44156_2023_18_MOESM2_ESM.bmp]

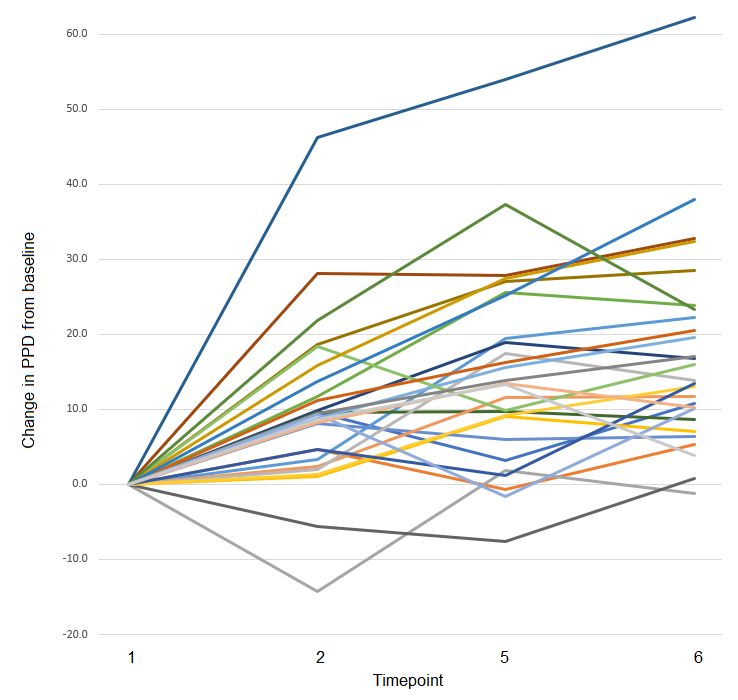

Supplement: Supplementary file 3 — Additional file 3: Figure S3. Change in Participant Packet Difference (PPD) from baseline for individual participants. [file 44156_2023_18_MOESM3_ESM.bmp]
