# Supplementary material for: Serial image interpretation tasks improve accuracy and increase confidence in Level 1 echocardiography reporting: a pilot study
Source: Echo Res Pract. 2023 Apr 6;10:6. doi: 10.1186/s44156-023-00018-9 (PMC10076813; doi:10.1186/s44156-023-00018-9)
Supplement: Supplementary file 4 — Additional file 4: Table S1. Summary of echocardiograms included in the study. AoV, Aortic Valve; AR, Aortic Regurgitation; LV, Left Ventricle; MR, Mitral Regurgitation; RV, Right Ventricle; RWMA, Regional Wall Motion Abnormality; TR, Tricuspid Regurgitation. [file 44156_2023_18_MOESM4_ESM.docx]

**Table S1. Summary of echocardiograms included in the study.**

| **Echo** | **Key Findings (finding scores 5-6/6)** | **Average score, %** | **Participants achieving perfect score,**  **n (%)** |
| --- | --- | --- | --- |
| A1-01 | *None (normal study)* | 79.1 | 4 (14.8) |
| A1-02 | LV hypertrophy, Pericardial Effusion | 76.2 | 1 (3.7) |
| A1-03 | Small LV, Dilated RV, Impaired RV, Significant TR | 83.3 | 4 (14.8) |
| A1-04 | LV Hypertrophy, Small LV, RV Hypertrophy, Dilated RV | 73.6 | 1 (3.7) |
| A1-05 | Impaired LV (RWMAs) | 75.9 | 1 (3.7) |
| A1-06 | Dilated RV, Impaired RV, Significant AR, Significant TR | 75.8 | 1 (3.7) |
| A1-07 | Small RV, Pericardial Effusion | 75.1 | 0 |
| A1-08 | *None (normal study)* | 78.3 | 4 (14.8) |
| A1-09 | Dilated LV, Impaired LV (RWMAs) | 61.7 | 0 |
| A1-10 | Impaired LV (global), Dilated RV, Impaired RV | 65.4 | 0 |
| A2-01 | LV Hypertrophy, Significant AR | 64.7 | 0 |
| A2-02 | Small LV, Dilated RV, Impaired RV, Significant TR | 85.6 | 3 (11.1) |
| A2-03 | *None (normal study)* | 88.9 | 7 (25.9) |
| A2-04 | LV Hypertrophy, RV Hypertrophy, Dilated RV, Impaired RV, Significant TR | 70.5 | 0 |
| A2-05 | Dilated LV, Impaired LV (RWMAs), Significant MR | 74.5 | 1 (3.7) |
| A2-06 | Impaired LV (RWMAs) | 75.6 | 1 (3.7) |
| A2-07 | LV Hypertrophy, Small LV | 68.1 | 1 (3.7) |
| A2-08 | *None (normal study)* | 66.5 | 0 |
| A2-09 | Dilated LV, Impaired LV (global), Dilated RV, Impaired RV | 66.9 | 0 |
| A2-10 | Dilated LV, Impaired LV (global), Impaired RV | 75.1 | 0 |
| B1-01 | Small LV, Dilated RV, Impaired RV, Significant TR | 83.5 | 2 (7.4) |
| B1-02 | *None (normal study)* | 71.8 | 1 (3.7) |
| B1-03 | Dilated LV, Impaired LV (global), Impaired RV, Significant AR | 74.8 | 1 (3.7) |
| B1-04 | Impaired LV (RWMAs), Impaired RV, Significant MR, Significant TR | 61.9 | 0 |
| B1-05 | Dilated RV, Impaired RV, Thickened AoV | 63.2 | 0 |
| B1-06 | LV Hypertrophy, Small LV | 675 | 0 |
| B1-07 | Pericardial Effusion | 74.2 | 2 (7.4) |
| B1-08 | LV impaired (global), Impaired RV, Significant TR | 67.2 | 0 |
| B1-09 | *None (normal study)* | 81.0 | 3 (11.1) |
| B1-10 | LV impaired (global) | 74.0 | 2 (7.4) |
| B2-01 | *None (normal study)* | 82.8 | 5 (18.5) |
| B2-02 | Small LV, Dilated RV, Impaired RV, Significant TR, Pericardial Effusion | 69.9 | 1 (3.7) |
| B2-03 | Dilated RV, Significant AR, Significant TR | 78.6 | 6 (22.2) |
| B2-04 | LV impaired (global), RV impaired, | 73.5 | 1 (3.7) |
| B2-05 | LV impaired (RWMAs) | 65.3 | 1 (3.7) |
| B2-06 | LV impaired (RWMAs) | 70.4 | 1 (3.7) |
| B2-07 | Dilated LV, Impaired LV (RWMAs), RV Hypertrophied, Thickened AoV | 63.3 | 0 |
| B2-08 | *None (normal study)* | 83.5 | 8 (29.6) |
| B2-09 | Significant MR, Significant TR | 81.9 | 2 (7.4) |
| B2-10 | LV Hypertrophy, Small LV, RV Impaired, Significant TR | 72.2 | 0 |

AoV, Aortic Valve; AR, Aortic Regurgitation; LV, Left Ventricle; MR, Mitral Regurgitation; RV, Right Ventricle; RWMA, Regional Wall Motion Abnormality; TR, Tricuspid Regurgitation.
